# Continuous-Flow Synthesis of Δ^9^-Tetrahydrocannabinol and Δ^8^-Tetrahydrocannabinol from Cannabidiol

**DOI:** 10.1021/acs.joc.3c00300

**Published:** 2023-04-04

**Authors:** Benedetta Bassetti, Christopher A. Hone, C. Oliver Kappe

**Affiliations:** † Institute of Chemistry, University of Graz, Heinrichstraße 28, A-8010 Graz, Austria; ‡ Center for Continuous Flow Synthesis and Processing (CCFLOW), Research Center Pharmaceutical Engineering GmbH (RCPE), Inffeldgasse 13, 8010 Graz, Austria

## Abstract

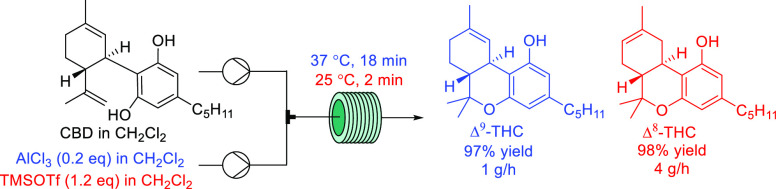

A challenging step in the preparation of tetrahydrocannabinol analogs is an acid-catalyzed intramolecular cyclization of the cannabidiol precursor. This step typically affords a mixture of products, which requires extensive purification to obtain any pure products. We report the development of two continuous-flow protocols for the preparation of (−)-*trans*-Δ^9^-tetrahydrocannabinol and (−)-*trans*-Δ^8^-tetrahydrocannabinol.

*Cannabis sativa* is an indigenous plant to Central Asia, which has been used for a variety of applications since ancient times.^1^ There are over 500 chemical entities that have been isolated from *Cannabis sativa*, of which the most representative class is phytocannabinoids, with over 120 isolated and characterized to date.^2^ Δ^9^-Tetrahydrocannabinol (Δ^9^-THC, **2**) is the primary psychoactive constituent of cannabis.^2^ Δ^9^-THC is a partial agonist at both cannabinoid receptor 1 (CB_1_), a modulator of psychoactive effects, and cannabinoid receptor 2 (CB_2_), a modulator of immunological and anti-inflammatory effects. Δ^9^-THC in its (−)-*trans* form is commercially available in the USA and Europe as dronabinol.^3^ Dronabinol is approved by the FDA for the treatment of HIV/AIDS-induced anorexia and chemotherapy-induced nausea and vomiting.

Under acidic conditions, Δ^9^-THC isomerizes to its thermodynamically more stable double bond isomer, Δ^8^-THC (**3**). Δ^8^-THC displays milder psychoactive effects when compared to Δ^9^-THC but shows comparable efficacy *in vitro* and *in vivo*.^4^ Although **3** is less abundant in natural cannabis than Δ^9^-THC, recently there has been growing interest in Δ^8^-THC.^5−8^

There are a number of synthetic strategies to prepare Δ^9^-THC.^9,10^ The first stereospecific preparation of (−)-Δ^9^-*trans*-THC (**2**) was reported by Mechoulam et al. in 1967.^11^ The synthesis occurs via the condensation of olivetol and (*S*)-*cis*-verbenol in the presence of a Lewis acid to afford the (−)-Δ^8^-*trans*-THC (**3**). **3** is then converted to **2** by treatment with hydrochloric acid and sodium hydroxide. Razdan et al. reported a one-step reaction from (+)-*trans*-*p*-mentha-2,8-dien-1-ol and olivetol in the presence of Lewis acid catalyst and MgSO_4_ to afford Δ^9^-THC in 31% yield after column chromatography.^12^ This method is still commonly used since **2** is formed in a single step. Recent methods have utilized asymmetric catalysis or auxiliaries for the stereospecific preparation of THC analogs.^13−15^ Nevertheless, considering scalability, the use of chiral pool feedstocks is favored, despite the fact that they provide low to moderate selectivity to a particular THC product.

The utilization of flow technologies for the synthesis of THC analogs has received recent interest due to the benefits provided in terms of control, efficiency, and scalability.^16−20^ Recently, Rutjes and co-workers reported continuous-flow synthesis methods from *p*-menthadienol and (−)-verbenol, but this afforded Δ^8^-THC and Δ^9^-THC in relatively low isolated yields, 17% and 30%, respectively.^19^ Antoniotti and co-workers reported a flow synthesis leading to the formation of truncated THC analogs but unfortunately with relatively poor selectivity.^20^

One strategy to form Δ^9^-THC and Δ^8^-THC is through the acid-catalyzed intramolecular cyclization of CBD.^21^ There are two main pathways, either via the activation of the Δ^8^ double bond to form Δ^9^-THC or the Δ^1^ double bond resulting in Δ^8^-*iso*-THC (**4**) (Table 1). Most studies focus on measuring the distribution of the reaction components at a single time end point.^22^ As both Δ^9^-THC and Δ^8^-*iso*-THC overreact to more thermodynamically stable products, Δ^8^-THC and (Δ^4^)^8^-*iso*-THC (**5**) respectively, this time data are of vital importance. There are contrasting results in the literature regarding the reaction of CBD in the presence of thermal and acidic conditions.^21,23,24^ The acid-catalyzed cyclization greatly depends on the reaction parameters, including the identity of the acid species, temperature, and reaction time.

**Table 1:**
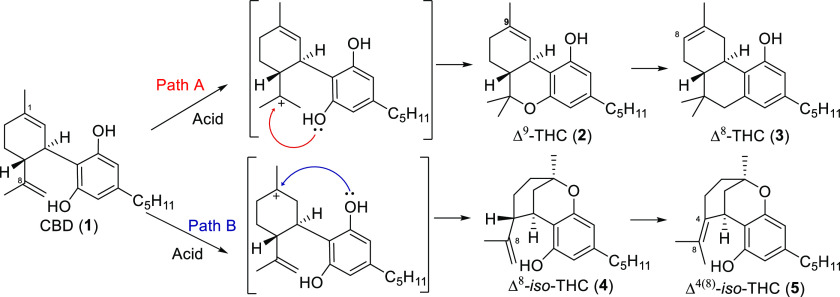
Acid Batch Screening of Acid-Catalyzed CBD (**1**) Cyclization^a^

entry	acid	*T* [°C]	time	conv. **1** [%]^b^	sel. **2** [%]^b^	sel. **3** [%]^b^	sel. **4** [%]^b^	sel. **5** [%]^b^
1	BF_3_·OEt_2_	–10	3.5 h	98	85	1	14	-
2	BF_3_·OEt_2_	0	1 h	>99	83	1	16	-
3^c^	BF_3_·OEt_2_	0	22 h	>99	1	54	7	32
4	TMSOTf	–10	2 min	97	81	13	5	-
5	TMSOTf	–10	1 h	>99	2	90	2	5
6	TMSCl	rt	48 h	63	83	3	14	-
7	In(OTf)_3_	–10	4 h	94	80	11	10	-
8	Sc(OTf)_3_	rt	3 h	98	81	13	6	-
9	TiCl_4_	–10	2 min	>99	11	43	2	2
10	AlCl_3_	–10	15 min	>99	87	2	3	-
11	*p*TSA	rt	1.5	93	68	28	2	-
12	CSA	40	3	81	67	30	4	-
13	TFA	0	3	88	77	17	6	-
14	HSO_3_Cl	–10	10 min	94	7	84	4	-

a Conditions: 0.1 M of **1** in CH_2_Cl_2_, 1.2 equiv of acid, and NaHCO_3_ as quench.

b Values determined by GC-FID peak area percent, and selectivity is percent of product with respect to all peaks except the substrate.

c PhMe as solvent.

In this study, we collected time profile data in batch under different acidic conditions and then used this knowledge to develop continuous-flow protocols for the selective synthesis of Δ^9^-THC and Δ^8^-THC. The influence of different Lewis and Brønsted acids on conversion and selectivity was explored (Table 1). As the acid species influences the reaction rate and selectivity, an understanding of the reaction kinetics is important to obtain high conversion of CBD while minimizing undesired products. A reaction network, consisting of four first-order rate-limiting steps based on the cannabinoid species, was considered (Figure 1). The four rate constants were fitted simultaneously for each profile using kinetic fitting software. The model structure was sufficient to describe the experimentally observed behavior for most acids.

**Figure 1:**
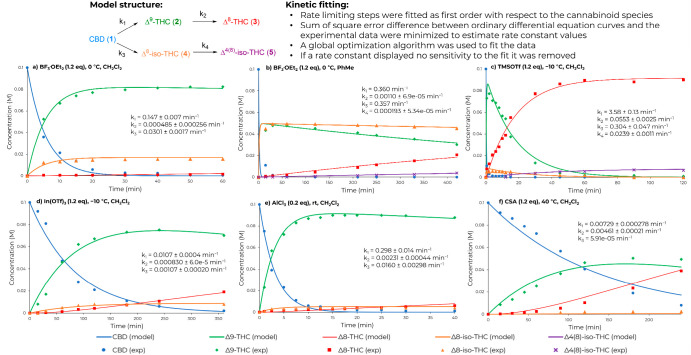
Kinetic fitting and reaction profiles under different acidic conditions.

Boron trifluoride etherate (BF_3_·OEt_2_) is a commonly used acid for the preparation of Δ^9^-THC from CBD. In our hands, BF_3_·OEt_2_ gave 98% conversion of CBD and 85% selectivity of Δ^9^-THC within 2 h at −10 °C (entry 1). The reaction rate displayed a 5-fold increase at 0 °C when compared to −10 °C (*k*_1,0 °C_/*k*_1,–10 °C_ = 5.09), providing 83% selectivity within 30 min at 0 °C (Figure 1a and Figure S2a). The overreaction of Δ^9^-THC to the more thermodynamically stable Δ^8^-THC isomer was very slow, with 1% selectivity. The main side product was Δ^8^-*iso*-THC, with a slight increase observed at 0 °C (entry 2). Interestingly, when switching to toluene (PhMe) as solvent, a higher formation of Δ^8^-*iso*-THC (**4**) was favored. After 22 h, the overreaction product, (Δ^4^)^8^-*iso*-THC (**5**), resulted in 32% selectivity (entry 3). The time profile (see Figure 1b) is particularly noteworthy, since the *iso*-THC compounds, **4** and **5**, have received less attention than Δ^9^-THC and Δ^8^-THC.^25^

Subsequently, we examined the use of trimethylsilyl trifluoromethanesulfonate (TMSOTf), which caused a rapid reaction rate (Figure 1c), with 97% conversion of CBD and 81% selectivity of Δ^9^-THC within 2 min of reaction time (entry 4). TMSOTf afforded 90% selectivity of Δ^8^-THC after 60 min of reaction time, and the overreaction of **4** to **5** was also observed (entry 5). This difference from 2 min to 1 h highlights the importance of collecting reaction profiles when developing an understanding of reaction selectivity. Trimethylsilyl chloride (TMSCl) was attempted but displayed a slow reaction rate, with only 63% conversion of **1** and 83% selectivity of Δ^9^-THC after 48 h at room temperature (entry 6). We next investigated the influence of metal triflates, In(OTf)_3_ and Sc(OTf)_3_. In(OTf)_3_ displayed moderate reactivity with 94% conversion of **1** and 80% selectivity of Δ^9^-THC within 4 h at −10 °C (entry 7 and Figure 1d). Sc(OTf)_3_ needed a higher temperature to promote the reaction and achieve a similar selectivity of Δ^9^-THC (entry 8).

The use of titanium chloride (TiCl_4_) resulted in a fast reaction rate with >99% conversion within 2 min but afforded a complex impurity profile (entry 9). To our delight, the use of aluminum trichloride (AlCl_3_) at −10 °C afforded full conversion of **1**, 87% selectivity of Δ^9^-THC, and low amounts of Δ^8^-THC and Δ^8^-*iso*-THC after 15 min of reaction (entry 10 and Figure 1e). This result is particularly interesting since no previous reports use AlCl_3_ for the preparation of Δ^9^-THC from CBD.

We then examined the influence of Bronsted acids. *p*-Toluene sulfonic acid (*p*TSA) needed 0 °C for activation, with 51% conversion of **1** and 80% selectivity of Δ^9^-THC after 30 h. Incomplete conversion (93%) was obtained at room temperature, with a selectivity of 68% and 28% for Δ^9^-THC and Δ^8^-THC, respectively (entry 11). In the case of camphorsulfonic acid (CSA), the use of low temperatures (−10 °C to rt) gave very low conversion and reaction rate. Under reflux conditions, 81% conversion of **1** and 67% selectivity of Δ^9^-THC were obtained (entry 12 and Figure 1f). However, after some investigation of the conditions, the product distribution between Δ^9^-THC and Δ^8^-THC could not be improved (Table S2). Trifluoroacetic acid (TFA) at −10 °C was selective to Δ^9^-THC but only provided 16% conversion of **1** after 6 h. 0 °C provided a higher reaction rate but gave a mixture of products (entry 13). The use of chlorosulfuric acid (HSO_3_Cl) as reagent resulted in a fast rate even at −10 °C, with 84% selectivity of Δ^8^-THC within 10 min.

We next considered the application of supported acid reagents (Table S4). Supported reagents and catalysts have the potential benefit that purification is simpler since the product is in a separate phase to the acid, assuming no leaching occurs.^26,27^ The promising results using BF_3_·OEt_2_ as reagent led us to investigate the reaction using silica-supported boron trifluoride (Si-BF_3_) and polyvinylpyrrolidone-supported boron trifluoride (PVP-BF_3_). The rate using Si-BF_3_ was substantially slower and less selective than for its homogeneous counterpart. 98% conversion of **1** and 65% selectivity of Δ^9^-THC were obtained after 6 h at 0 °C. A peak in the GC chromatogram observed at 12.1 min retention time was assigned to the aromatized oxidation product, cannabinol (CBN, **S1**). Si-BF_3_ followed the same trend as when using homogeneous BF_3_·OEt_2_ as reagent, with more Δ^8^-*iso*-THC formed at higher temperatures. The reaction rate could be significantly increased by operating at room temperature with no drop in selectivity toward Δ^9^-THC. When using PVP-BF_3_, a high yield of Δ^9^-THC could be obtained after a prolonged reaction time (156 h) at room temperature. 99% conversion of **1** and 89% selectivity of Δ^9^-THC were achieved after 6 h at 40 °C, which, although lower in reactivity, is similar to the selectivity achieved using standard BF_3_·OEt_2_. Due to the promising results with Sc(OTf)_3_, scandium on polymer was attempted, but only trace amounts (<1%) of Δ^9^-THC were observed.

Polymer-bound *p*TSA displayed higher activity than standard *p*TSA, with 73% conversion of **1** after 27 h at −10 °C. In a similar manner to *p*TSA, the use of polymer-bound *p*TSA also afforded a mixture. The reaction rate could be increased at higher temperatures, but not the product distribution to favor Δ^9^-THC. We also investigated silica-propylsulfonic acid, Amberlyst 15, and Nafion NR50, but all resulted in a mixture of products. Nafion NR50 at room temperature still resulted in incomplete conversion (81%) even after 8 days, probably due to the relatively small surface area of the Nafion NR50 solid.^28^ Gratifyingly, the reaction with Montmorillonite K10 (MK10) at room temperature proceeded with 98% conversion of **1** and high selectivity (84%) of Δ^9^-THC after 5 h.

We next turned our attention to the development of flow protocols for the selective preparation of Δ^9^-THC and Δ^8^-THC. We selected five of the acids which had been demonstrated to be the most promising from the batch screening: MK10, PVP-BF_3_, BF_3_·Et_2_O, TMSOTf, and AlCl_3_. Our goal was to develop a protocol that provided high selectivity toward either Δ^9^-THC or Δ^8^-THC within a short residence time in a reproducible and scalable manner.

For the supported acid flow configuration, a single syringe pump was used for the introduction of **1** in CH_2_Cl_2_, which then flowed through a packed bed of the supported acid catalyst contained within a column cartridge (Scheme 1a). We observed promising results with MK10 when operating in flow, with 81% yield of Δ^9^-THC obtained within 1.5 min at room temperature. However, when fractionating the outlet, the performance of the system changed over the run time, and different results were obtained after packing the column with fresh MK10. Previously, the use of MK10 for the reaction of **1** in flow was reported, but there was no demonstration of long-term stability.^29^ Our results are consistent with observations made by others regarding the inconsistent performance of commercial MK10.^30^ This variation could be caused by the availability of acid sites depending on the packing of the clay, and water content within the clay can impact performance. A consistent reactor performance was not achieved when performing long run experiments using PVP-BF_3_. The conversion of **1** and yield of Δ^9^-THC displayed a linear decrease over the duration of the runs (Figure S5). Unfortunately, the supported acid catalysts did not show sufficient stability. The deactivation of solid acids is a significant disadvantage which limits their synthetic applicability.

**Scheme 1:**
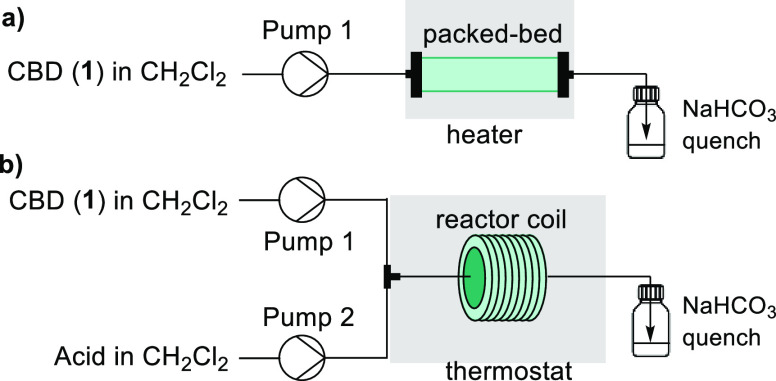
(a) Solid-Supported Flow Configuration and (b) Two-Feed Flow Configuration

The two-feed continuous flow setup consisted of syringe pumps, one for the introduction of **1** and a second pump for the introduction of the acid solution (Scheme 1b). The two feeds were combined within a tee-piece before entering the reactor coil. A challenge in batch is that careful addition of the acid is necessary to prevent an exotherm, whereas isothermal conditions are maintained in flow when the two streams mix. The timing of the quench was achieved by introducing the effluent in a semibatch manner to a vessel containing NaHCO_3_.

BF_3_·OEt_2_ in flow afforded Δ^9^-THC and Δ^8^-*iso*-THC in 83% and 15% selectivity, respectively, within 15 min of residence time at 10 °C. These results were fairly consistent to our batch data, but further investigations could not decrease the high amounts of Δ^8^-*iso*-THC formed. In the case of TMSOTf, the batch results showed that precise control over reaction time was necessary to maintain control over the selectivity toward Δ^9^-THC, due to the rapid overreaction to Δ^8^-THC. On the other hand, we did identify that a selective synthesis of Δ^8^-THC could be achieved in a 2 min residence time by operating at room temperature, providing 91% selectivity (87% assay NMR yield).^31^ We performed a 4.77 mmol scale experiment for the preparation of Δ^8^-THC for a run time of 24 min (Figure S9). The system performed in a stable manner and afforded Δ^8^-THC in 98% yield after filtration and removal of CH_2_Cl_2_.

We knew that we would need to optimize the AlCl_3_ equivalents to avoid solubility issues in flow. Thus, we performed further batch optimization and identified that good performance could be achieved with 0.2 equiv of AlCl_3_ by raising the reaction temperature (Figure 1e and Table S5). A peristaltic pump was used for handling the AlCl_3_ feed as it can handle suspensions. At the optimized conditions, the reaction was performed using 18 min residence time and 37 °C (Table S10). A long run experiment, corresponding to a 13.3 mmol scale, was performed over a total operation time of 470 min to demonstrate the robustness of the protocol. The system performed consistently for the duration of the run (Figure S8). >99% conversion of **1** and 92% selectivity (90% assay NMR yield) of Δ^9^-THC were observed for the combined fractions. After filtration and removal of CH_2_Cl_2_, Δ^9^-THC was obtained in 97% isolated yield, which corresponds to a throughput of 1.02 g/h.^31^

In summary, we have developed continuous flow protocols for the preparation of Δ^9^-THC and Δ^8^-THC from CBD in high yields. The conventional synthetic protocols afford a mixture of products which are difficult to purify. The use of flow takes advantage of the ability to precisely control reaction parameters which have a critical influence over product selectivity. The approaches described herein drastically improve the reaction performance. We believe this new approach will expand the interest in the use of flow technologies for the synthesis of THC analogs in the future.

## Data Availability

The data underlying this study are available in the published article and its online Supporting Information.

## Supporting Information Available

Description and images of the experimental setups, optimization data, and ^1^H NMR and ^13^C{^1^H} NMR of isolated products (PDF)
