# Supplementary material for: Continuous-Flow Synthesis of Δ9-Tetrahydrocannabinol and Δ8-Tetrahydrocannabinol from Cannabidiol
Source: J Org Chem. 2023 Apr 4;88(9):6227–31. doi: 10.1021/acs.joc.3c00300 (PMC10167683; doi:10.1021/acs.joc.3c00300)
Supplement: Supplementary file 1 — jo3c00300_si_001.pdf [file jo3c00300_si_001.pdf]

Supporting Information

Continuous-Flow Synthesis of Δ^9-Tetrahydrocannabinol and Δ^8-Tetrahydrocannabinol from Cannabidiol

Benedetta Bassetti,[†] Christopher A. Hone*[†,‡] and C. Oliver Kappe*[,†,‡]

[†] Institute of Chemistry, University of Graz, NAWI Graz, Heinrichstrasse 28, A-8010 Graz, Austria

[‡] Center for Continuous Flow Synthesis and Processing (CCFLOW), Research Center Pharmaceutical Engineering GmbH (RCPE), Inffeldgasse 13, 8010 Graz, Austria

*E-mail: christopher.hone@rcpe.at; oliver.kappe@uni-graz.at

Table of Contents

1 General Information 3
1.1 Materials and Methods 3
1.2 High Field NMR 3
1.3 GC-FID Analysis 3
1.4 GC-MS Analysis 3
1.5 Typical GC-FID Chromatogram 5
2 Batch Experiments 6
2.1 Acid Screening 6
2.1.1 Lewis Acids 7
2.1.2 Brønsted (Protic) Acids 8
2.1.3 Supported Acids 9
2.2 $AlCl_3$ Further Batch Optimization 12
3 Continuous Flow Investigation 13
3.1 General Flow Configurations 13
3.1.1 Montmorillonite K10 / PVP-BF_3 13
3.1.2 $AlCl_3$ 13
3.2 Flow Experiments 14
3.2.1 TMSOTf 14
3.2.2 $BF_3 \cdot OEt_2$ 15
3.2.3 PVP-BF_3 16
3.2.4 Montmorillonite K10 17
3.2.5 $AlCl_3$ 17
3.3 Long Run to Prepare Δ^9-THC 17
3.4 Preparative-scale Experiment to Prepare Δ^8-THC 21
4 Kinetic Fitting 24
5 Compound Characterization from Long Runs 25
6 NMR Spectra (^1H NMR and $^{13}C\{^1H\}$ NMR) 27

1 General Information

1.1 Materials and Methods

All materials were obtained from commercial suppliers (TCI, Sigma Aldrich, Alfa Aesar or VWR) and used without further purification unless otherwise noted. 5'-methyl-4-pentyl-2'-(prop-1-en-2-yl)-1',2',3',4'-tetrahydro-[1,1'-biphenyl]-2,6-diol (CBD, **1**) was obtained from Lonza Group AG and used without further purification. PVP-BF_3 was prepared as described in: Bloemendal, V. R. L. J. et al. *J. Flow Chem.* **2021**, *11*, 99–105. Dichloromethane (99.9% extra dry) was used for reactions.

1.2 High Field NMR

NMR spectra of final compounds were recorded on a Bruker 300 MHz instrument. ^{1}H and $^{13}C\{^{1}H\}$ spectra were recorded at 300 MHz and 75 MHz, respectively, with a chemical shift relative to TMS expressed in parts per million (ppm). The samples were prepared in $CDCl_3$. The letters s, d, t and m are used to indicate singlet, doublet, triplet, and multiplet, respectively.

1.3 GC-FID Analysis

GC-FID analysis was performed on a Shimadzu GC FID 230 with a flame ionization detector, using an RTX-5MS column (30 m × 0.25 mm ID × 0.25 μm) and helium as carrier gas (40 cm/sec linear velocity). The injector temperature was set to 280 °C. After 1 min at 50 °C, the temperature was increased by 25 °C/min to 300 °C and kept constant at 300 °C for 4 min. The detector gases used for flame ionization were hydrogen and synthetic air (5.0 quality). A representative GC chromatogram is provided below, see Section 1.5.

1.4 GC-MS Analysis

GC-MS analysis was performed using a Shimadzu GCMS-QP2010 SE, using an RTX-5MS column (30 m × 0.25 mm × 0.25 μm) and helium as carrier gas with a linear velocity of 40 cm/sec. The injector temperature was set to 280 °C. After 1 min at 50 °C, the oven temperature was increased by 25 °C/min

to 300 °C and then kept at 300 °C for 3 min. The mass detector was a quadrupole with pre rods and electron impact ionization. The following settings were used in the detector: ion source temperature 200 °C, interface temperature 310 °C, solvent cut time 2 min 30 sec, acquisition mode scan, mass range m/z = 50 till m/z = 400.

Note: In the case of GC and GC/MS care should be taken to ensure that the analysis is representative of the reaction and that decomposition does not occur due to the analytical method.

In the study by Tsujikawa et al., they investigated the thermal decomposition of CBD to Δ^9-THC by GCMS.[1] They identified that decomposition was not observed when using split mode, under any condition. We used split mode and we also confirmed that no decomposition occurs by injecting samples of standards for CBD, Δ^9-THC and Δ^8-THC under our GC-MS and GC-FID and we did not see any decomposition products.

THC and CBD exist as tetrahydrocannabinol acid (THCA) and cannabidiol acid (CBDA) in their natural form within the plant. The main advantage of HPLC is that it can identify the acidic components, THCA and CBDA, before conversion to their corresponding free forms of THC and CBD.[2] The main limitation of GC is that it does not detect THCA or CBDA directly, since CBDA and THCA are thermally labile decarboxylates to THC and CBDA decarboxylates to CBD.[2,3] However, THCA and CBDA are not relevant in our study, since we are investigating a chemical synthesis from CBD. These acidic cannabinoid components are not relevant when studying a chemical synthesis for the acid-catalyzed cyclization of CBD. Moreover, Marzullo et al. performed acid screening for this reaction using HPLC and NMR analysis and did not observe these compounds.[4]

References

1. Tsujikawa, K.; Okada, Y.; Segawa, H. Yamamuro, T.; Kuwayama, K.; Kanamori, T.; Iwata, Y. T. Thermal decomposition of CBD to Δ9-THC during GC-MS analysis: A potential cause of Δ9-THC misidentification. Forensic Sci. Int. 2022, 337, 111366.
2. For a discussion of HPLC and GC techniques, see: Pourseyed Lazarjani, M.; Torres, S.; Hooker, T.; Fowlie, C.; Young, O.; Seyfoddin, A. Methods for quantification of cannabinoids: a narrative review. *J. Cannabis Res.* **2020**, *2*, No. 35.
3. https://resources.perkinelmer.com/lab-solutions/resources/docs/app_cannabis-analysis-potency-testing-identifification-and-quantification-011841b_01.pdf, Received 13th January 2023.
4. For a study of the acid-catalyzed cyclization of CBD which used HPLC and NMR, see: Marzullo, P.; Foschi, F.; Coppini, D. A.; Fanchimi. F.; Magnani, L.; Rusconi, S.; Luzzani, M.; Passarella,

D. Cannabidiol as the Substrate in Acid-Catalyzed Intramolecular Cyclization. *J. Nat. Prod.* **2020**, *83*, 2894–2901.

1.5 Typical GC-FID Chromatogram

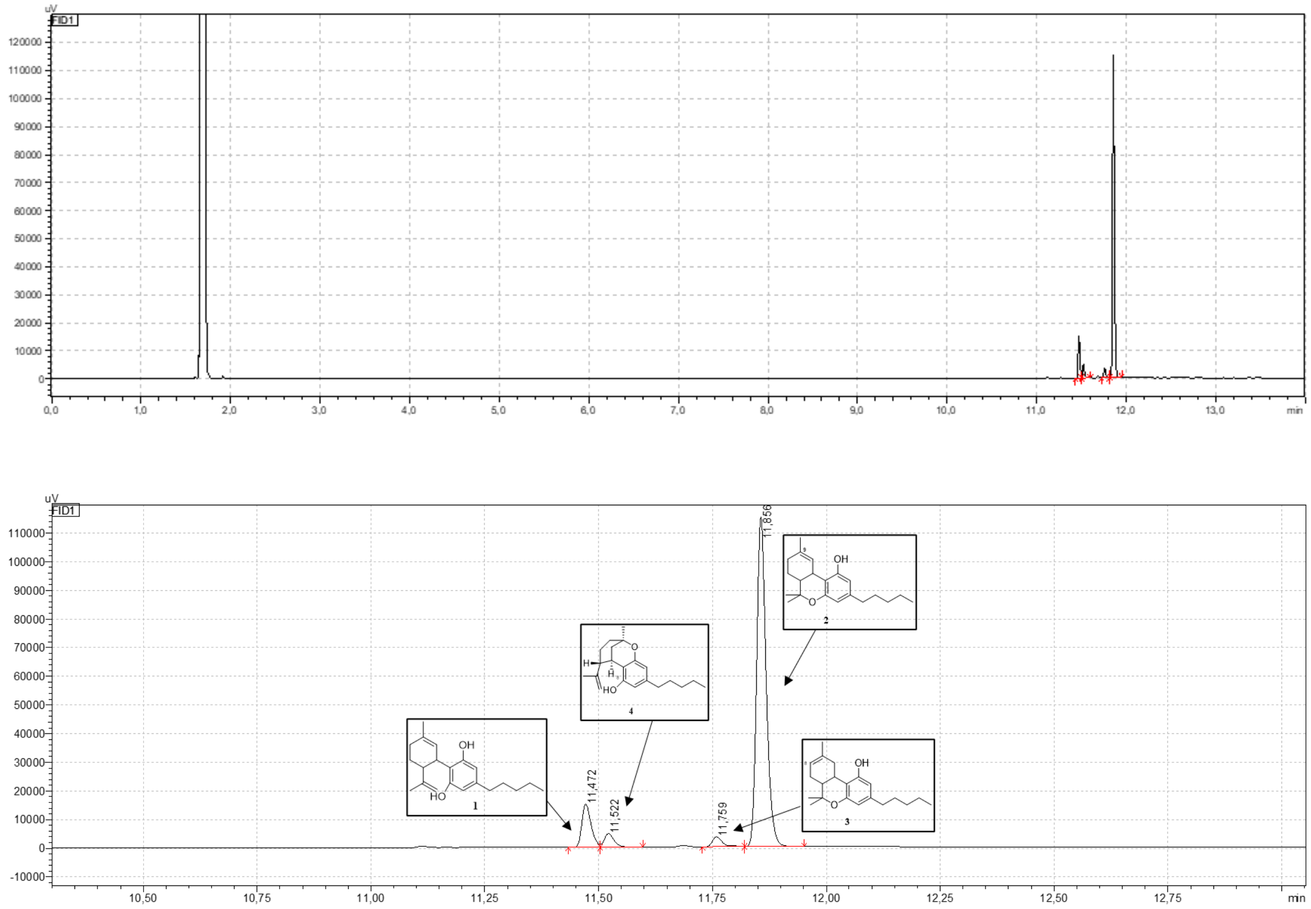

Figure S1. Representative GC-FID chromatogram of the acid-catalyzed cyclization of CBD (**1**), example 1: full chromatogram (above), zoomed-in (below).

2 Batch Experiments

2.1 Acid Screening

We investigated the acid-catalyzed cyclization of CBD (**1**) by studying the influence of different Lewis and Brønsted acids on the reaction performance (Scheme S1). A time profile was collected for each experiment. Initially we screened each acid reagent at −10 °C and then operate at higher temperatures when necessary to promote the reaction or increase the reaction rate.

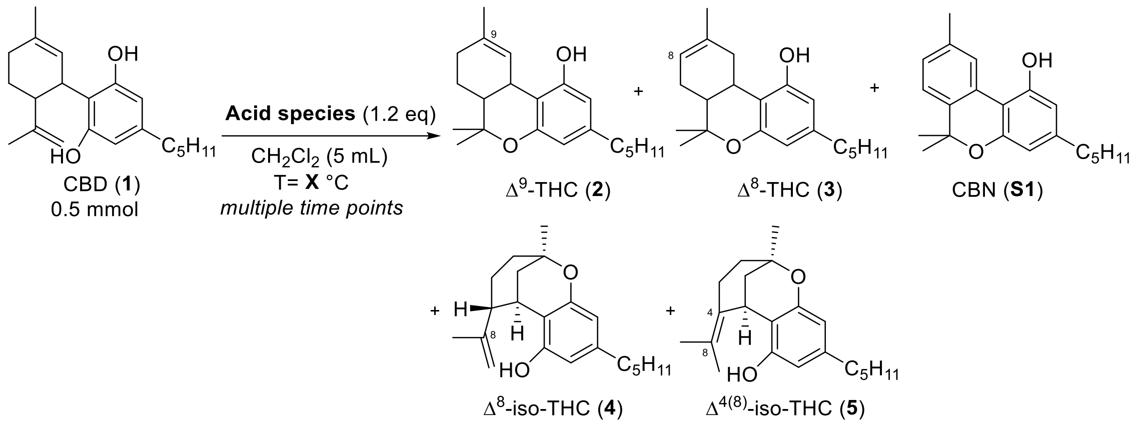

Scheme S1. General reaction scheme for the screening of Brønsted and Lewis acids via batch profiling. The general conditions are stated within the scheme and the experimental procedure is described below.

An exemplary batch procedure is described below. If not otherwise noted, batch experiments were executed using this procedure.

Substrate **1** (0.157 g, 0.500 mmol) was dissolved in anhydrous dichloromethane (5 mL, 0.1 M). The reaction was maintained at the desired temperature and the solution was stirred (minimum 500 rpm) using a magnetic stirrer. Then the acid (1.2 eq) was added and the reaction was profiled over time. Aliquots were taken from the reaction and added to a saturated solution of $NaHCO_3$ to quench the reaction. Then an aliquot was taken for analysis: 25 µL reaction mixture was diluted in 975 µL dichloromethane and analyzed by GC analysis.

2.1.1 Lewis Acids

Table S1. Selected results for the measured responses from the Lewis acid screening. Conditions reported in Scheme S1 were used unless otherwise stated.

entry	acid	T [°C]	time	conv. **1** [%][a]	sel. **2** [%][a]	sel. **3** [%][a]	sel. **4** [%][a]	sel. **5** [%][a]
1	$BF_3{\cdot}OEt_2$	−10 °C	3.5 h	98	85	1	14	-
2	$BF_3{\cdot}OEt_2$	0	1 h	>99	83	1	16	-
3[b]	$BF_3{\cdot}OEt_2$	0	7 h	>99	30	21	45	4
4[b]	$BF_3{\cdot}OEt_2$	0	22 h	>99	1	54	7	32
5	$In(OTf)_3$	−10	4 h	94	80	11	10	-
6	$In(OTf)_3$	0	30 min	88	81	9	10	-
7	$Sc(OTf)_3$	0	30 h	83	92	5	4	-
8	$Sc(OTf)_3$	rt	3 h	98	81	13	6	-
9	TMSOTf	−10	2 min	97	81	13	5	-
10	TMSOTf	−10	1 h	>99	2	90	2	5
11	TMSCl	RT	48 h	63	83	3	14	-
12	$TiCl_4$	−10	2 min	>99	11	43	2	2
13	$AlCl_3$	−10	15 min	>99	87	2	3	-

[a] Determined by GC-FID peak area percent. Percent of product with respect to all peaks except the substrate. [b]Reaction was performed in PhMe as solvent. RT = room temperature.

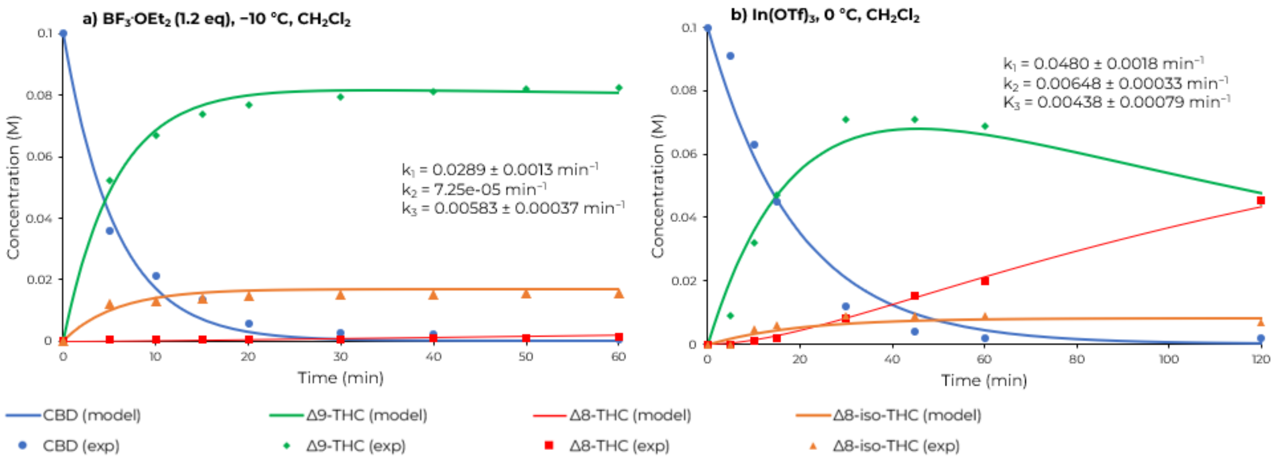

Figure S2. Reaction profiles and kinetic fitting for $BF_3{\cdot}OEt_2$ and $In(OTf)_3$. See also Fig. 1 in main manuscript.

2.1.2 Brønsted (Protic) Acids

Table S2. Selected results for the measured responses from the Brønsted acid screening. Values are determined by GC-FID. Conditions reported in Scheme S1 were used unless otherwise stated.

entry	acid	T [°C]	time	conv. **1** [%][a]	sel. **2** [%][a]	sel. **3** [%][a]	sel. **4** [%][a]
1	*p*TSA	0	30 h	51	80	8	8
2	*p*TSA	RT	1.5 h	93	68	28	2
3	CSA	40	3 h	81	67	30	4
4	CSA	65 [MW]	45 min	49	80	16	4
5[b]	CSA	90 [MW]	15 min	81	59	37	4
6[b]	CSA	110 [MW]	10 min	96	23	74	3
7	TFA	−10 °C	6 h	16	94	6	-
8	TFA	0	3 h	88	77	17	6
9	HSO_3Cl	−10 °C	10 min	94	7	84	4

[a]Determined by GC-FID peak area percent. Percent of product with respect to all peaks except the substrate. [b]Experiments were performed using 1.5 eq of acid. No overreaction of Δ^8-*iso*-THC (**4**) to form $(\Delta^4)^8$-*iso*-THC (**5**) was observed in the presence of Brønsted acids. RT = room temperature. MW = microwave irradiation.

2.1.2.1 CSA Microwave General Procedure

CBD (**1**) (0.078 g, 0.25 mmol) was dissolved in anhydrous dichloromethane (2.5 mL, 0.1 M) in a 5 mL microwave vial equipped with a stirring bar. CSA (1.2 eq) was added to the vial and it was sealed. The microwave vial was placed in Anton Parr Monowave 400 reactor at the desired temperature and time (see Table S2, entries 4-6). After the reaction, the microwave vial was cooled into an ice/NaCl bath, and a saturated solution of $NaHCO_3$ was added to quench the reaction. An aliquot of the reaction was taken for analysis: 25 µL reaction mixture was diluted in 975 µL dichloromethane and analyzed by GC analysis.

2.1.3 Supported Acids

2.1.3.1 Supported Acids General Batch Procedure

CBD (**1**) (0.157 g, 0.5 mmol) was dissolved in anhydrous dichloromethane (5 mL, 0.1 M). The reaction flask was immersed in an ice/NaCl bath to keep the temperature at −10 ° C. Subsequently, the supported acid was added in the desired amount (see below). The reaction was stirred (minimum 500 rpm) using a magnetic stirrer and controlled to the desired temperature. An aliquot of the reaction was taken for analysis: 25 µL reaction mixture was diluted in 975 µL dichloromethane and analyzed by GC analysis.

Table S3. Selected results for the measured responses from the supported acid screening.

entry	Acid (loading)	T [°C]	time	conv. **1** [%][a]	sel. **2** [%][a]	sel. **3** [%][a]	sel. **4** [%][a]
1[b]	*p*-TSA on polymer (200 mg)	−10	27 h	73	70	12	10
2[b]	*p*-TSA on polymer (200 mg)	RT	45 min	87	55	32	10
3[b]	Si-propylsulfonic acid (983 mg)	−10	6 h	25	88	4	-
4[b]	Si-propylsulfonic acid (983 mg)	0	4 h	85	68	13	7
5[b]	Amberlyst 15 (200 mg)	0	6 h	69	72	12	9
6[b]	Amberlyst 15 (200 mg)	RT	2 h	88	55	31	9
7	Mont. K10 (200 mg)	0	4 h	89	12	-	-
8	Mont. K10 (200 mg)	RT	5 h	98	84	3	8
6	Si-BF_3 (200 mg)	−10	4 h	92	68	1	12
7	Si-BF_3 (200 mg)	0	6 h	98	65	4	14
8	Si-BF_3 (200 mg)	RT	10 min	95	72	3	20
9	PVP-BF_3 (400 mg)	RT	156 h	>99	89	4	7
10	PVP-BF_3 (400 mg)	40	6 h	99	84	3	13

[a]Determined by GC-FID peak area percent. Percent of product with respect to all peaks except the substrate. RT = room temperature. [b]No overreaction of Δ^8-*iso*-THC (**4**) to form $(\Delta^4)^8$-*iso*-THC (**5**) was observed.

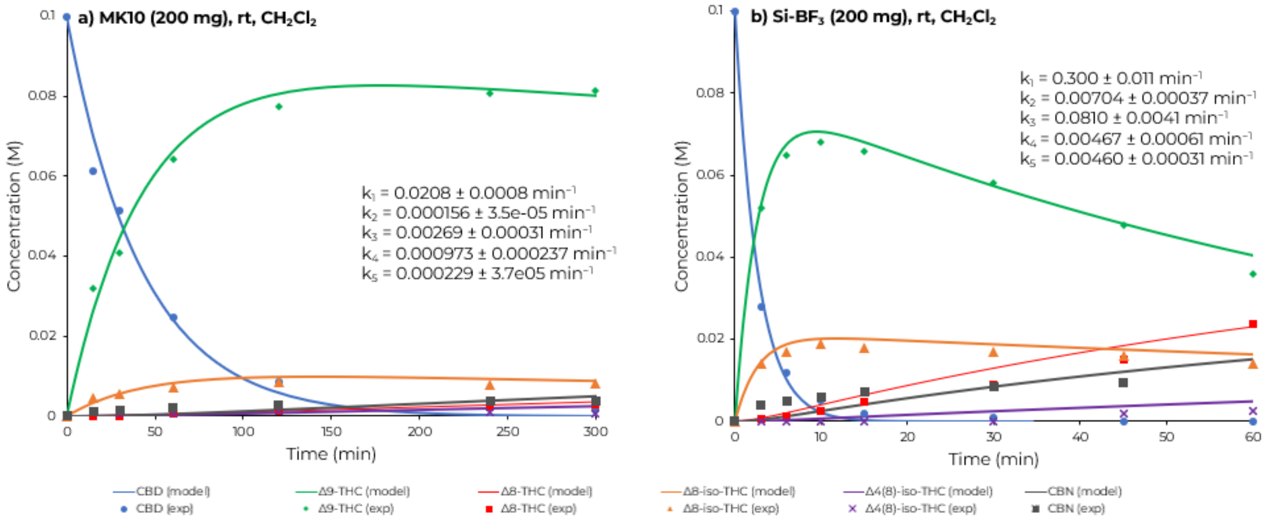

Figure S3. Reaction profiles and kinetic fitting for MK10 and Si-BF3.

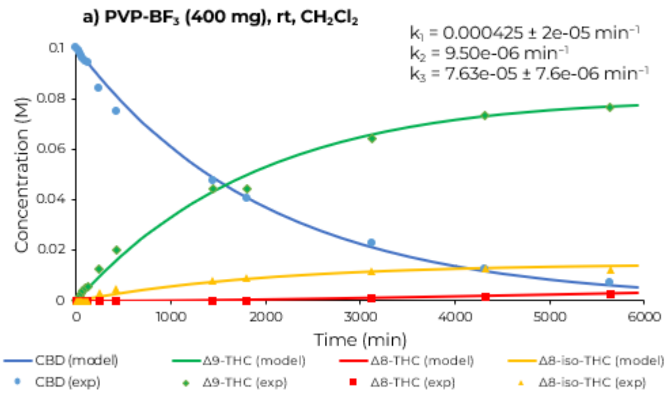

Figure S4. Reaction profiles and model fitting for PVP-BF3.

Table S4. Best results from batch screening of supported acids.

entry	acid	loading	T [°C]	time	conv. **1** [%][a]	sel. **2** [%][a]	sel. **3** [%][a]	sel. **4** [%][a]
1	Si-BF_3	200 mg	0	6 h	98	65	4	14
2	Si-BF_3	200 mg	rt	10 min	95	72	3	20
3	PVP-BF_3	400 mg	rt	156 h	>99	89	4	7
4	PVP-BF_3	400 mg	40	6 h	99	84	3	13
5	*p*TSA on polymer	240 mg	rt	45 min	87	55	32	10
6	Si-propylsulfonic acid	983 mg	0	4 h	85	68	13	7
7	Amberlyst 15	200 mg	0	6 h	69	72	12	9
8	Nafion NR50	400 mg	rt	8 days	81	38	22	11
9	MK10	200 mg	rt	5 h	98	84	3	8

Conditions: 0.1 M of **1** in CH_2Cl_2 and $NaHCO_3$ used as quench. [a]Values determined by GC-FID peak area percent and selectivity is percent of product with respect to all peaks except the substrate.

2.2 $AlCl_3$ Further Batch Optimization

Table S5. Results for the measured responses for the batch optimization using $AlCl_3$.

entry	acid eq	solvent	T [°C]	time [min]	conv. **1** [%][a]	sel. **2** [%][a]	sel. **3** [%][a]	sel. **4** [%][a]
1	1.2	CH_2Cl_2	−10	15	>99	88	3	3
2	1.2	CH_2Cl_2	−10	10	>99	86	8	3
3	1.2	PhMe	−10	20	>99	78	5	8
4	1.2	PhMe/Et_2O	−10	60	>99	81	4	14
5	0.5	CH_2Cl_2	0	15	>99	89	4	4
6	0.1	CH_2Cl_2	0	120	93	95	2	3
7	0.1	CH_2Cl_2	RT	30	90	92	3	4
8	0.2	CH_2Cl_2	RT	25	98	92	4	4

General conditions: All experiments were performed using 0.5 mmol of **1** in 5 mL of solvent. [a] Determined by GC-FID peak area percent. Percent of product with respect to all peaks except the substrate. No overreaction of Δ^8-*iso*-THC (**4**) to form $(\Delta^4)^8$-*iso*-THC (**5**) was observed. RT = room temperature.

3 Continuous Flow Investigation

3.1 General Flow Configurations

For pumping feed solutions, syringe pumps (Syrris Asia) equipped with syringes appropriate for the desired flow rate were used. All of the pumps were used with check valves (Upchurch, CV-3321) and internal pressure sensors. The pressure limit of the pumps was set to 20 bar. The pumps would turn-off automatically for safety reasons above this pressure. The syringe pumps were calibrated by pumping for a specified time and checking the mass balance. All pumps were found to dose within ± 2%. Standard PFA tubing (0.8 mm or 1.6 mm i.d.), PTFE or PEEK fittings and T-pieces were used in the flow setups. The reactor coil was cut to length depending on the desired volume required. The reactor coil was kept at the desired temperature (−20 to 40 °C) by placing it inside the heating fluid which was maintained at a constant temperature using a thermostat (Huber Ministat 230).

We selected five acids from the screening to continue our investigations in: TMSOTf, $BF_3{\cdot}Et_2O$, $AlCl_3$, PVP-BF_3 and Montmorillonite K10. We investigated the influence of changing substrate concentration, acid equivalents, residence time and temperature.

3.1.1 Montmorillonite K10 / PVP-BF_3

Montmorillonite K10 or PVP-BF_3 was packed into a column (Omnifit) and then placed on the heater module (Syrris Asia) to perform the reaction at the desired temperature.

3.1.2 $AlCl_3$

For pumping light suspensions, i.e., in the case of $AlCl_3$, a peristaltic pump (Vapourtec SF-10) was used. Above this pressure and the pump would turn-off automatically for safety reasons.

3.2 Flow Experiments

3.2.1 TMSOTf

Table S6. Measured responses for the flow optimization experiments using TMSOTf.

Entry	CBD (**1**) [M]	Acid equiv	T [°C]	t_{res} [min]	conv. **1** [%][a]	sel. **2** [%][a]	sel. **3** [%][a]	sel. **4** [%][a]	sel. **5** [%][a]
1	0.2	1.2	−10	2	95	3	94	1	-
2	0.2	0.6	−10	1	93	74	18	5	-
3	0.1	0.1	−10	1	31	90	3	6	-
4	0.1	0.5	−10	1	90	84	7	6	-
5	0.05	0.5	−10	1	>99	81	14	5	-
6	0.05	0.5	−20	1	88	88	5	5	-
7	0.05	0.5	−20	1.5	97	86	9	5	-
8	0.2	1.2	0	4	97	19	75	4	-
9	0.2	1.2	0	8	>99	3	89	2	6
10	0.2	1.2	25	2	>99	2	91	1	5
11	0.2	1.2	25	4	>99	2	89	1	6
12	0.2	1.5	25	2	>99	2	89	0	6
13	0.2	2	30	2	>99	2	88	1	6
14	0.2	1.2	25	2	>99	2	91	1	5

[a] Determined by GC-FID peak area percent. Percent of product with respect to all peaks except the substrate.

3.2.2 $BF_3 \cdot OEt_2$

Table S7. Measured responses for the flow optimization experiments using $BF_3 \cdot Et_2O$.

Entry	Acid equiv	t_{res} [min]	T [°C]	conv. **1** [%][a]	sel. **2** [%][a]	sel. **3** [%][a]	sel. **4** [%][a]
1	1.2	15	0	95	78	1	21
2	2	15	5	97	84	2	14
3	2	15	10	>99	83	2	15
4	0.5	5	RT	95	79	2	19

Experiments were performed using 0.1 M solution of CBD. [a] Determined by GC-FID peak area percent. Percent of product with respect to all peaks except the substrate.

3.2.3 PVP-BF_3

Table S8. Measured responses for a flow experiment using PVP-BF_3.

Entry	Acid [mg]	t_{res} [min]	T [°C]	**1** [%][a]	**2** [%][a]	**3** [%][a]	**4** [%][a]
1	500	7	40	12-40	50-71.5	1.5-7	7.5-11.5

Experiment was performed using 0.1 M solution of CBD (**1**). [a]Determined by GC-FID peak area percent.

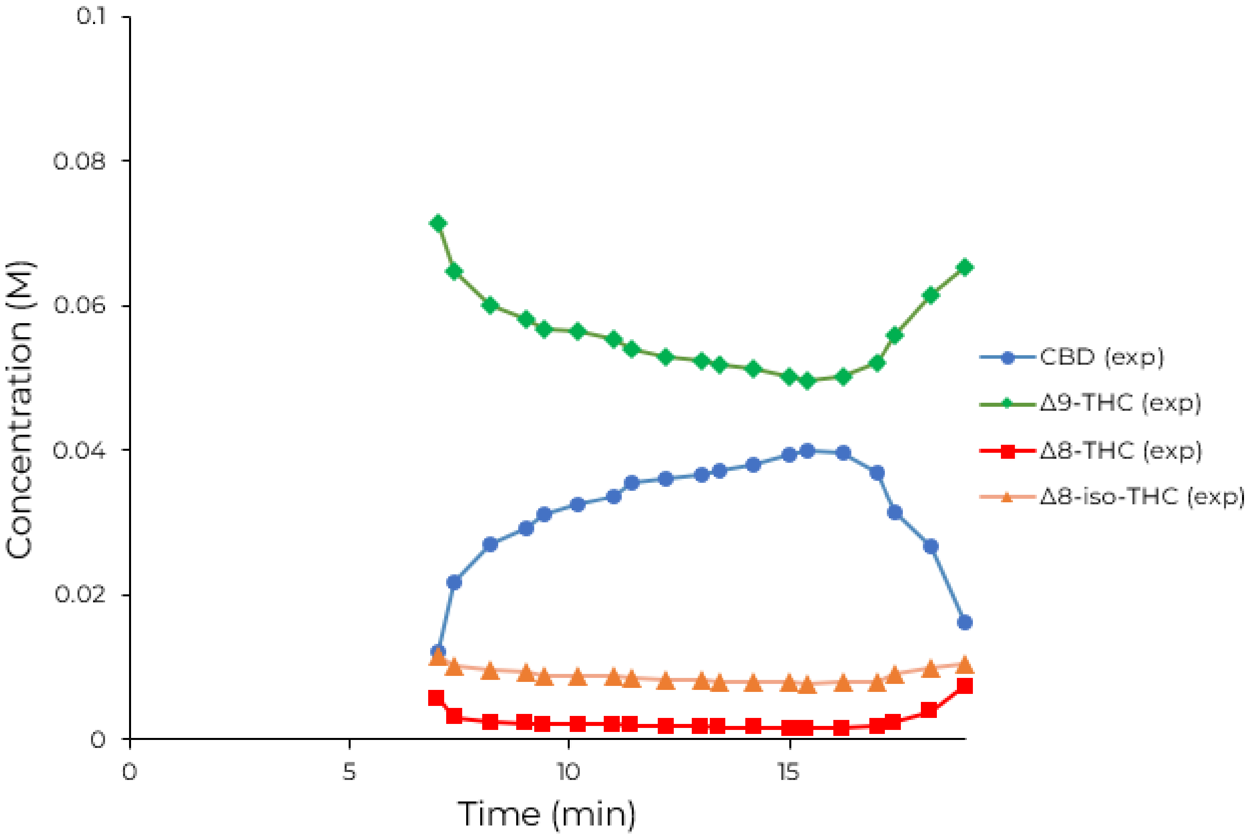

Figure S5. Table S8 Entry 1: PVP-BF_3, reaction profile over time.

We attempted different residence times in order to achieve constant activity over run time, however the general trends did not change. Thus, we abandoned this acid since we were never able to reach steady-state conditions.

3.2.4 Montmorillonite K10

Table S9. Measured responses for the flow experiments using Montmorillonite K10. Values are determined by GC-FID. All experiments are performed at RT using 0.1 M solution of CBD.

Entry	Acid [mg]	t_{res} [min]	conv. **1** [%][a]	sel. **2** [%][a]	sel. **3** [%][a]	sel. **4** [%][a]
1	405	2	99	80	7	13
2[b]	405	2	91	86	2	12
3	405	1.5	94	86	2	12
4[c]	405	1.5	99	80	8	12

Experiment was performed using 0.1 M solution of CBD (**1**). [a] Determined by GC-FID peak area percent. Percent of product with respect to all peaks except the substrate. [b]Entry 1 repeated, but using fresh clay. [c]Entry 3 repeated.

3.2.5 $AlCl_3$

Table S10. Results for the measured responses for the flow optimization using $AlCl_3$.

Entry	T [°C]	t_{res} [min]	conv. **1** [%][a]	sel. **2** [%][a]	sel. **3** [%][a]	sel. **4** [%][a]
1	20	16.66	90	93	2	4
2	25	16.66	95	92	3	5
3	25	20	87	93	2	5
4	25	18	97	91	3	6
5	30	18	99	90	3	7
6	35	18	97	92	3	5
7	37	18	99	92	4	4

General conditions: All experiments were performed using 0.2 M feed solution of CBD (**1**) and 0.04 M feed solution of $AlCl_3$. [a]Determined by GC-FID peak area percent. Percent of product with respect to all peaks except the substrate.

3.3 Long Run to Prepare Δ^9-THC

We performed a long run for the preparation of Δ^9-THC (**2**) using the optimized conditions shown in Table S10, entry 7, to assess the robustness of our protocol.

Before/after experiment:

Before running the reactions, the system was flushed with technical grade dichloromethane for 10 min. After the experiments, the setup was rinsed with dichloromethane and then the system was stored under isopropanol.

Feed preparation:

Feed solutions were prepared in volumetric flasks. *Substrate feed preparation:* Cannabidiol (**1**) (6.28 g, 20.0 mmol) was dissolved in anhydrous dichloromethane (100 mL, 0.2 M). *Acid feed preparation:* Aluminium trichloride ($AlCl_3$) (1.06 g, 8 mmol) was dissolved in anhydrous dichloromethane (200 mL, 0.04 M).

Flow reaction protocol:

The system was operated for a total of 268 min (from start-up to collecting the final fraction), which corresponded to the processing 4.20 g (13.3 mmol) of CBD (**1**). An image of the flow setup used can be seen in Fig. S7. The feed solution of $AlCl_3$ (0.04 M) in CH_2Cl_2 was introduced using a peristaltic pump (Vapourtec SF-10). The $AlCl_3$ feed solution was stirred at 37 °C for the duration of the experimental run. CBD (0.2 M) in CH_2Cl_2 was introduced using a syringe pump (Syrris Asia). The substrate solution was pumped at a flow rate of 0.278 mL/min and the $AlCl_3$ solution was pumped at a flow rate of 0.278 mL/min, giving a residence time of 18 min. A simple T-piece was used to mix the two feeds prior to the reactor. The reactor coil (10 mL internal volume) was submerged in the thermostat heating solution (EtOH) to control the temperature, which was set at 37 °C. The outlet stream was fractionated into stirred vials containing $NaHCO_3$ in CH_2Cl_2. Sample collection was started once color was observed at the end of the reactor (after 18 min). The reaction outlet was collected in 10 mL vials containing a quench ($NaHCO_3$ in CH_2Cl_2) and a stirring bar, the first 30 min (1 vial every 10 min), then in 20 mL vials for the central 3.5 h (1 vial every 30 min), and again in a 10 mL vial for the last ten minutes. Eleven fractions were collected and analyzed on the GC-FID (Table S11). 1,2-dichloro-4-nitrobenzene was used as internal standard to measure the NMR assay yield, which was determined to be 90% (value based on the average of three separate NMR samples). The collected fractionated vials were combined, filtered and then solvent was removed under reduced pressure (the rotary evaporator bath was maintained at 35 °C) to obtain **2** (4.07 g, 12.9 mmol, 97% yield) as a yellow oil (Fig. S6). The yellow oil was characterized by ^{1}H NMR, $^{13}C\{^{1}H\}$ NMR and GC-MS (Section 5).

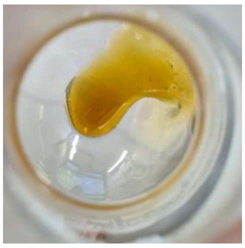

Figure S6. Δ^{9}-THC (**2**).

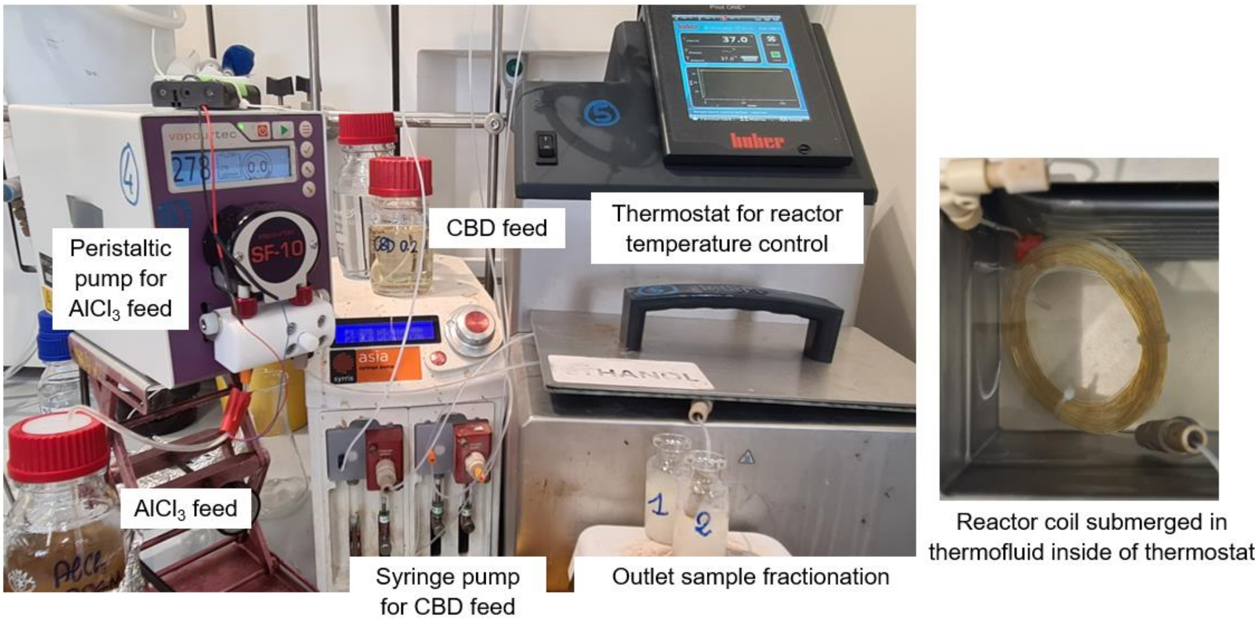

Figure S7. Labeled image of continuous-flow setup for the preparation of Δ^{9}-THC (**2**).

Table S11. Measured responses for the Δ[9]-THC (**2**) long run. The reaction was fractionated into vials during the 268 min run time.

vial	fractionated time interval [min]	**1** [%]	**2** [%]	**3** [%]	**4** [%]
1	18-28	1.2	91.6	2.4	4.7
2	28-38	1.2	91.8	2.1	4.9
3	38-48	1.1	91.7	2.1	5.0
4	48-78	0.9	91.9	2.0	5.0
5	78-108	1.0	91.9	2.1	5.0
6	108-138	1.0	92.0	1.9	5.1
7	138-168	1.0	91.5	2.0	5.1
8	168-198	0.8	91.6	1.9	5.1
9	198-228	0.9	91.6	2.0	5.0
10	228-258	0.7	91.9	2.0	4.9
11	258-268	0.7	91.9	2.0	4.9
Average		1.0	91.8	2.1	5.0

[a]Responses were determined by GC-FID.

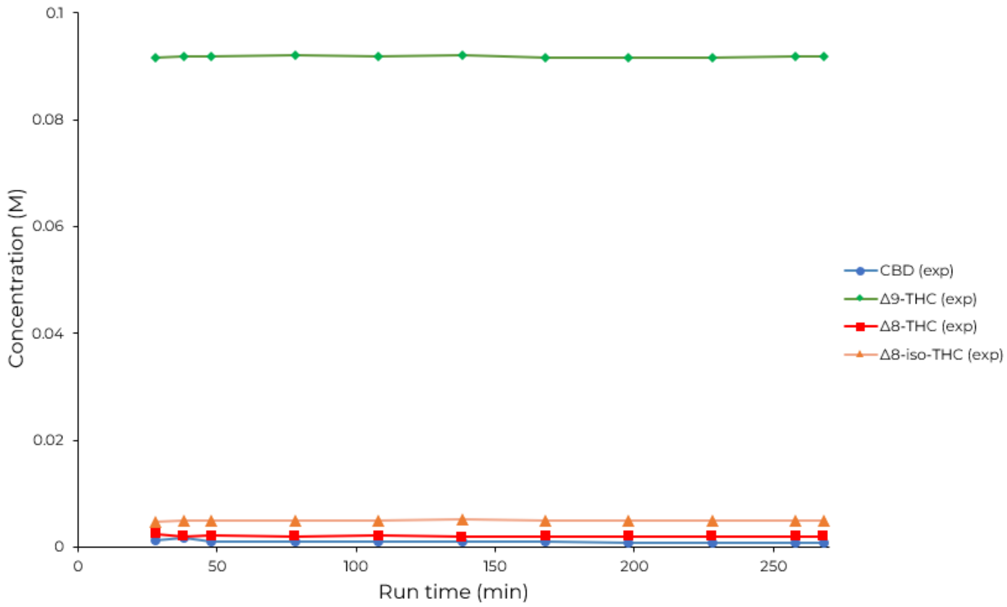

Figure S8. Concentration of the different components over the duration of the long run for the preparation of Δ[9]-THC (**2**).

3.4 Preparative-scale Experiment to Prepare Δ^8-THC

We performed an experiment for the preparation of Δ^8-THC (**3**) using the optimized conditions shown in Table S6 Entry 14, to assess the robustness of our protocol.

Before/after experiment:

Before running the reactions, the system was flushed with technical grade dichloromethane for 10 min. After the experiments, the setup was rinsed with dichloromethane and then the system was stored under isopropanol.

Feed preparation:

Feed solutions were prepared in volumetric flasks. *Substrate feed preparation:* Cannabidiol (**1**) (1.51 g, 4.80 mmol) was dissolved in anhydrous dichloromethane (24 mL, 0.2 M). *Reagent feed preparation:* Trimethylsilyl trifluoromethanesulfonate (TMSOTf) (1.28 g, 1.05 mL, 5.76 mmol) was dissolved in anhydrous dichloromethane (24 mL, 0.24 M).

Flow reaction protocol:

The system was operated for a total of 24 min (from start-up to collecting the final fraction). The two feed solutions were introduced at the same flow rate using syringe pumps (Syrris Asia). The combined flow rate was 2.25 mL/min. A simple T-piece was used to mix the two feeds prior to the reactor. The reactor coil (4.5 mL internal volume) was submerged in the thermostat heating solution (EtOH) to control the temperature, which was set at 25°C. The reaction outlet was collected in 10 mL vials containing a quench ($NaHCO_3$ in CH_2Cl_2) and a stirring bar, for the first 3 min (1 vial every 1 min), then in 10 mL vials for the central 16 min (1 vial every 2 min), and again in a 4 mL vial for the last three min. Fourteen fractions were collected and analyzed on the GC-FID (Table S12). 1,2-dichloro-4-nitrobenzene was used as internal standard to measure the NMR assay yield, which was determined to be 87% (value based on the average of three separate NMR samples). The collected fractionated vials were combined, filtered and then solvent was removed under reduced pressure (the rotary evaporator bath was maintained at 35 °C) to obtain **3** (1.48 g, 4.71 mmol, 98% yield) as a red oil (Fig. S10). The red oil was characterized by ^{1}H NMR and ^{13}C{^{1}H} NMR (Section 5).

Table S12. Measured GC yields for the Δ^8-THC (**3**) preparative-scale experiment. The reaction was fractionated into vials during the 24 min of run time.

vial	fractionated time interval [min]	**1** [%][a]	**2** [%][a]	**3** [%][a]	**4** [%][a]	**5** [%][a]
1	2-3	-	2.1	90.8	-	4.3
2	3-4	-	2.2	89.8	-	4.7
3	4-5	-	2.2	90.2	-	4.7
4	5-7	-	1.9	90.7	--	4.4
5	7-9	-	2.0	90.2	-	4.5
6	9-11	-	2	90.1	-	4.5
7	11-13	-	2.1	90.5	-	4.5
8	13-15	-	1.9	90.9	-	4.4
9	15-17	-	1.9	90.8		4.5
10	17-19	-	1.9	91	-	4.4
11	19-21	-	2.0	90.2	-	4.6
12	21-22	-	2.2	90.0	-	4.3
13	22-23	-	1.9	90.9	-	4.1
14	23-24	-	2.1	91.2	0.3	4.3
Average		-	2.0	90.5	0.0	4.5

[a]Responses were determined by GC-FID.

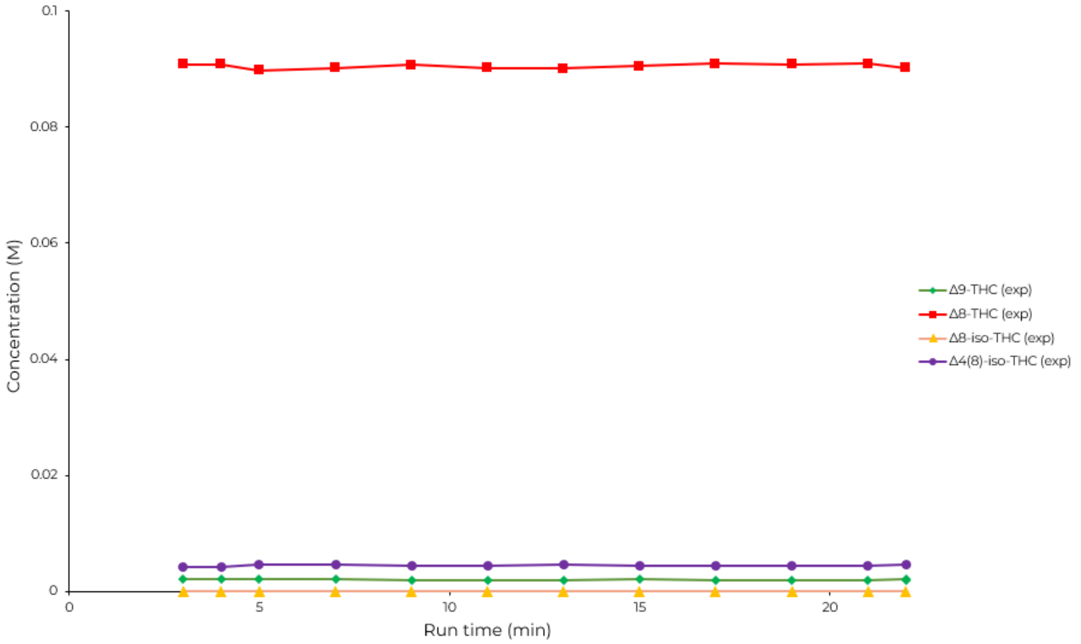

Figure S9. Concentration of the different components over the duration of the experiment for the preparation of Δ^8-THC (**3**).

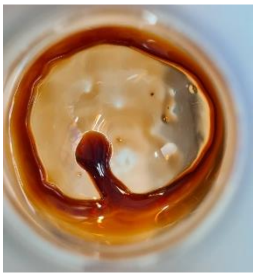

Figure S10. Δ^8-THC (**3**).

4 Kinetic Fitting

The fitting of the rate constants for the rate limiting steps was performed using a software package called Compunetics (v3.1.1) (https://compunetics.net/). The software automatically adjusted the rate constant values based to improve the model fit to the experimental data. The sum of squared error (SSE) difference between the ordinary differential equation (ODE) curves and the experimental data were minimized to arrive at the final estimated rate constant values. The errors on fitted rate constant values are absolute errors, whereby this is the maximum possible error in the ODE based on fittings of each permutation of the ±2.5% relative error specified concentration for each of the species fitted. Moreover, the maximum variance was determined from the original fitting, and reported as the absolute error in the parameter fit. If a rate constant displayed no sensitivity to the fit then it was removed. The model structure which was fitted to the reaction profiles is shown in Scheme S2. The four rate limiting steps were simultaneously fitted for the reaction profile. The reactions were fitted as first order. In the case of the batch reaction with MK10 and Si-BF_3 an additional rate-limiting step, k_5, Δ^9-THC (**2**) to CBN (**S1**) was also fitted (Fig. S3).

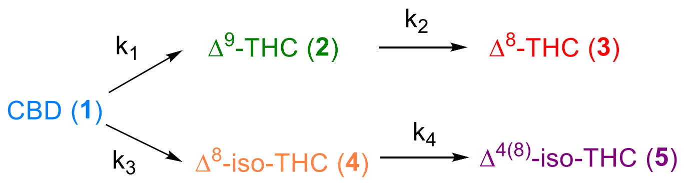

Scheme S2. Model structure for the fitting of the rate constants.

5 Compound Characterization from Long Runs

(−)-*trans*-Δ⁹-Tetrahydrocannabinol (Δ⁹-THC, 2)[1,2]

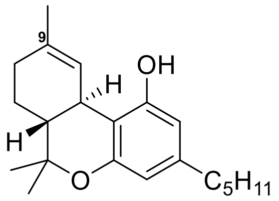

Δ^9-THC (**2**)

Yellow oil

^{1}H NMR (300 MHz, $CDCl_3$) δ 6.33 (s, 1H), 6.30 (d, *J* = 1.2 Hz, 1H), 6.16 (d, *J* = 1.3 Hz, 1H), 4.81 (s, 1H), 3.26-3.19 (m, 1H), 2.46 (dt, *J* = 7.4, 2.2 Hz, 2H), 2.23-2.16 (m, 2H), 1.97-1.89 (m, 1H), 1.74-1.65 (m, 4H), 1.58 (m, 2H), 1.47-1.40 (m, 4H), 1.37-1.27 (m, 4H), 1.12 (s, 3H), 0.90 (t, *J* = 6.9 Hz, 3H).

^{13}C{^{1}H} NMR (75 MHz, $CDCl_3$): δ 154.7, 154.2, 142.8, 134.4, 123.7, 110.1, 109.1, 107.5, 45.8, 35.5, 33.6, 31.5, 31.2, 30.6, 27.6, 25.0, 23.4, 22.5, 19.3, 14.0.

GC-MS analysis: m/z 314 confirmed

(−)-*trans*-Δ⁸-Tetrahydrocannabinol (Δ⁸-THC, 3)[1,2]

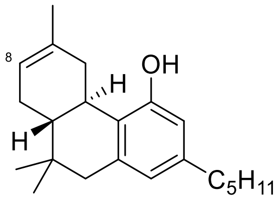

Δ^8-THC (**3**)

Red oil

^{1}H NMR (300 MHz, $CDCl_3$) δ 6.30 (s, 1H), 6.13 (brs, 1H), 5.45 (m, 1H), 3.22 (dd, *J* = 15.7, 3.1 Hz, 1H), 2.72 (td, *J* = 10.8, 4.7 Hz, 1H), 2.46 (td, *J* = 7.5, 3.3 Hz, 2H), 2.20-2.13 (m, 1H), 1.91-1.79 (m, 2H), 1.73 (s, 3H), 1.62-1.55 (m, 2H), 1.41 (s, 3H), 1.38-1.29 (m, 5H), 1.14 (s, 3H), 0.91 (t, *J* = 6.9 Hz, 3H). 1,2-dichloro-4-nitrobenzene was used as internal standard to assess the purity of the yellow oil which was measured to be 87% (value based on the average of three separate NMR samples).

^{13}C{^{1}H} NMR (75 MHz, $CDCl_3$): δ 154.9, 154.7, 142.7, 134.7, 119.3, 110.5, 110.2, 107.6, 77.6, 44.9, 36.0, 35.5, 31.6, 30.6, 27.9, 27.6, 23.5, 22.5, 18.5, 14.0.

GC-MS analysis: m/z 314 confirmed.

References:

1. Cheng, L.-J.; Xie, J.-H.; Chen, Y.; Wang, L.-X.; Zhou, Q.-L. Enantioselective Total Synthesis of (−)-Δ^8-THC and (−)-Δ^9-THC via Catalytic Asymmetric Hydrogenation and S_NAr Cyclization. *Org. Lett.* **2013**, *15*, 764–767.
2. Fahrenholtz, K. E.; Lurk, M.; Kierstead, R. W. The Total Synthesis of *dl*-Δ9-Tetrahydrocannabinol and Four of Its Isomers. *J. Am. Chem. Soc.* **1967**, *89*, 5934–5941.

6 NMR Spectra (^{1}H NMR and ^{13}C{^{1}H} NMR)

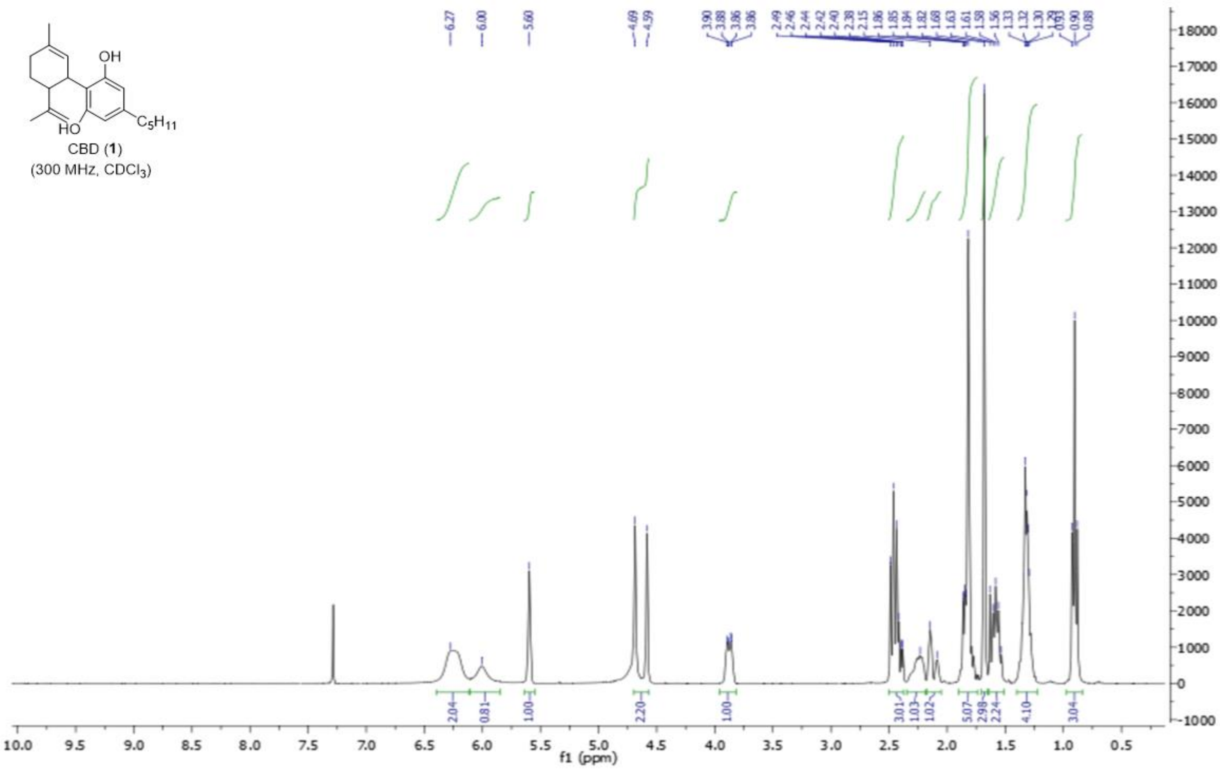

^{1}H NMR Spectrum of Starting Material CBD (**1**)

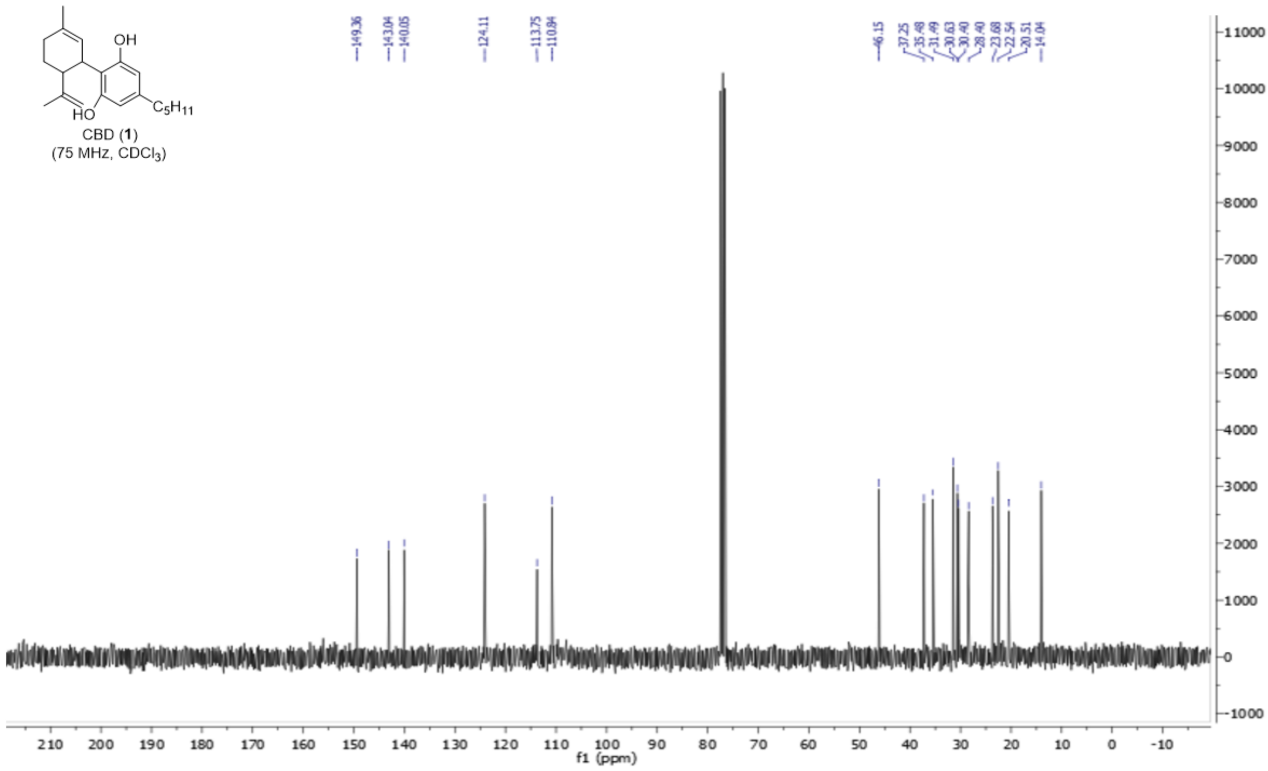

^{13}C{^{1}H} NMR Spectrum of Starting Material CBD (**1**)

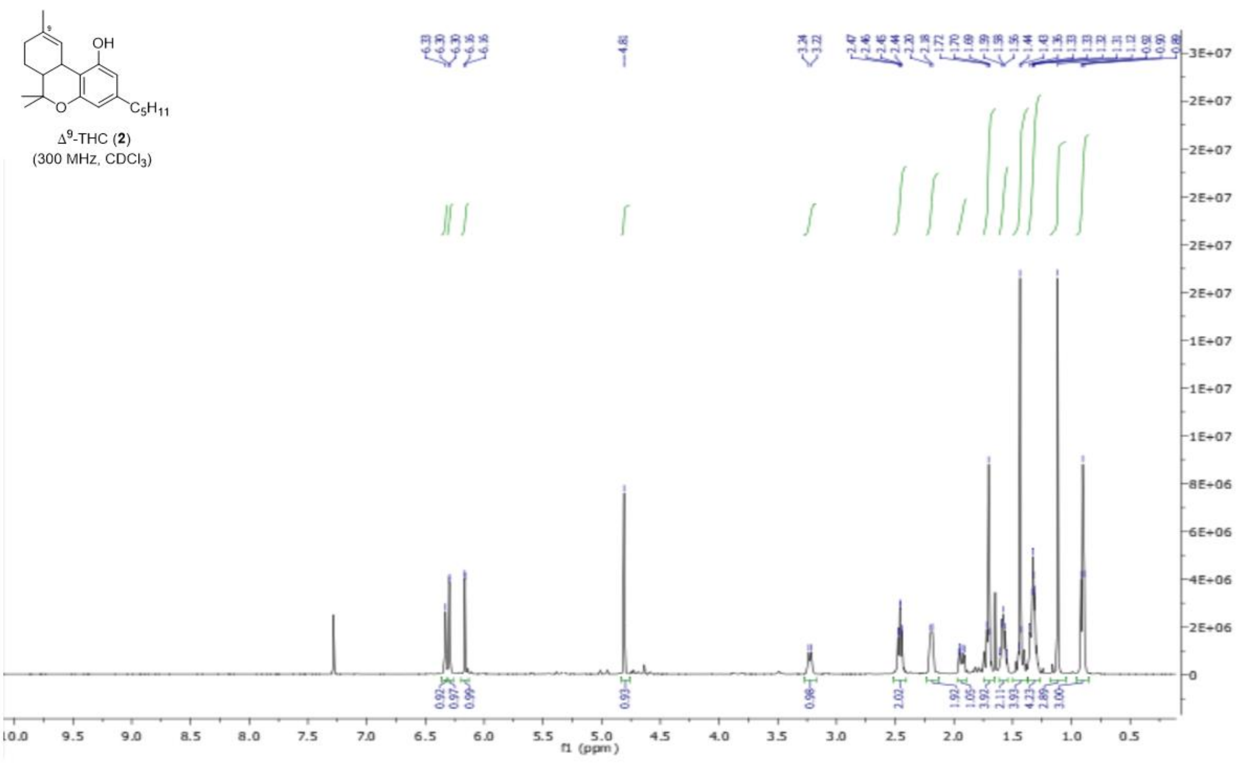

^{1}H NMR Spectrum of *trans*-Δ^9-THC (**2**)

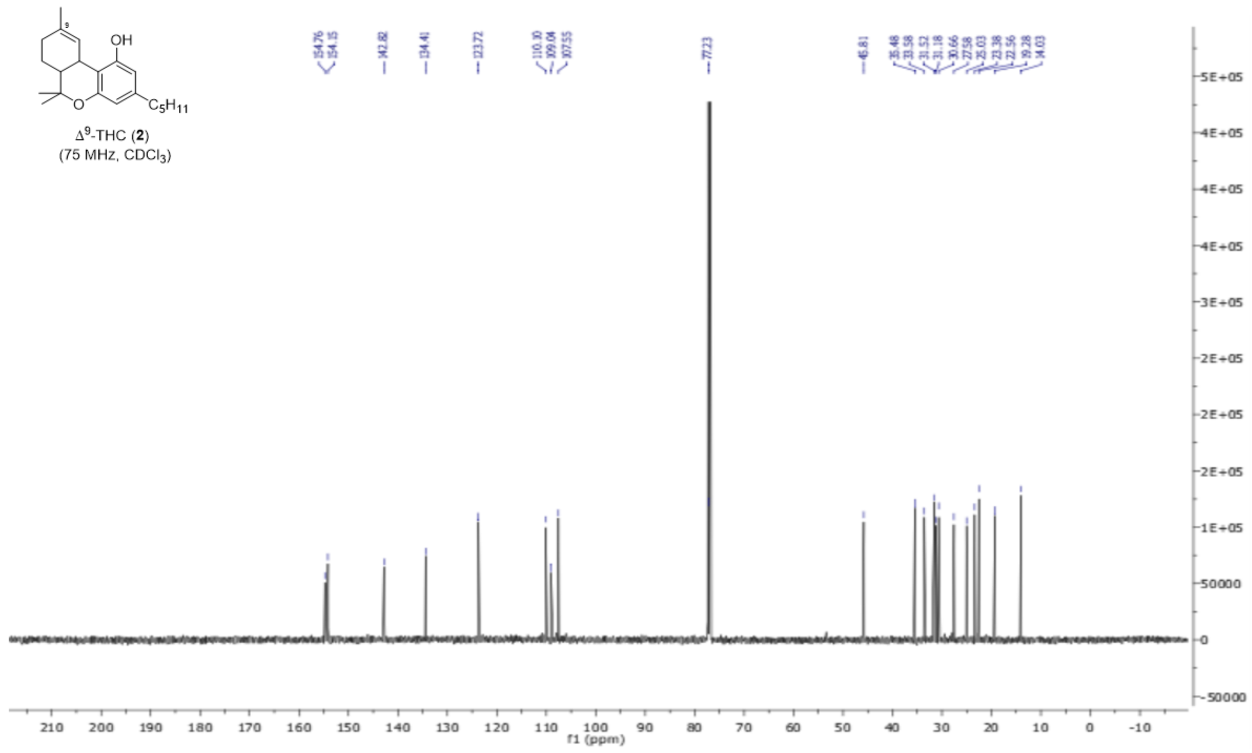

^{13}C{^{1}H} NMR Spectrum of *trans*-Δ^9-THC (**2**)

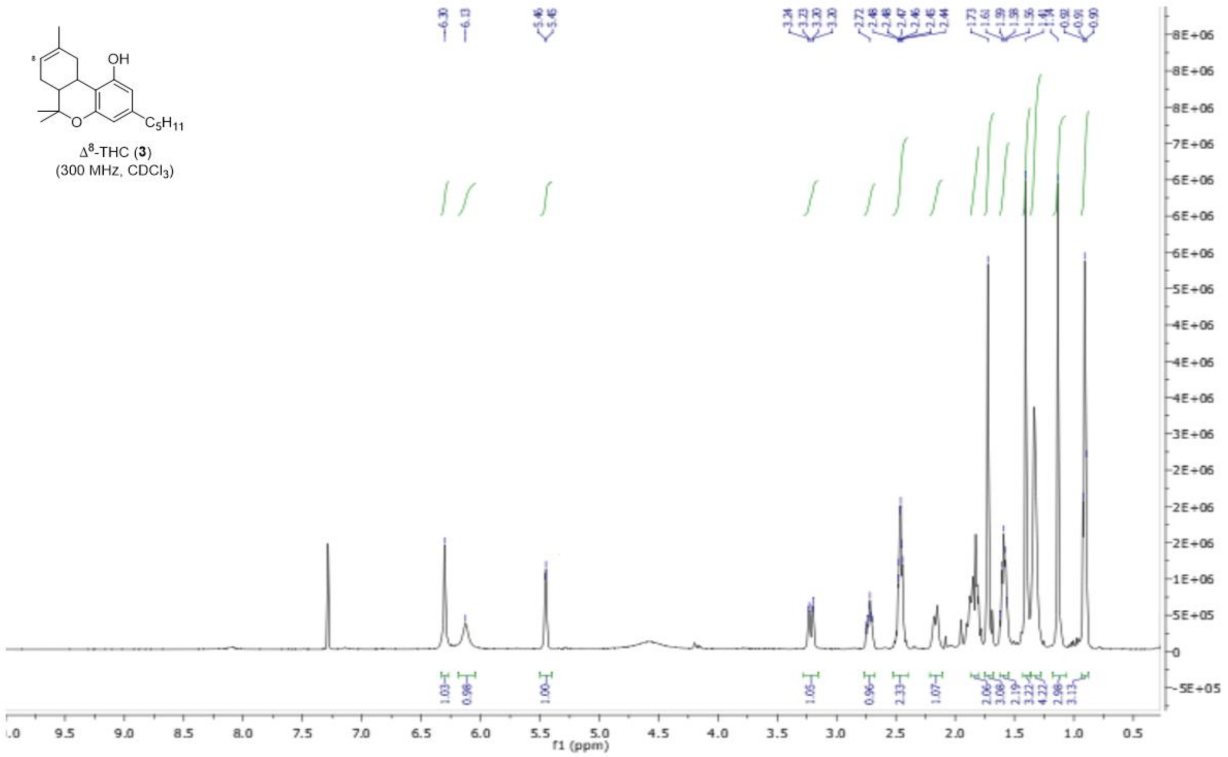

^{1}H NMR Spectrum of *trans*-Δ^8-THC (**3**)

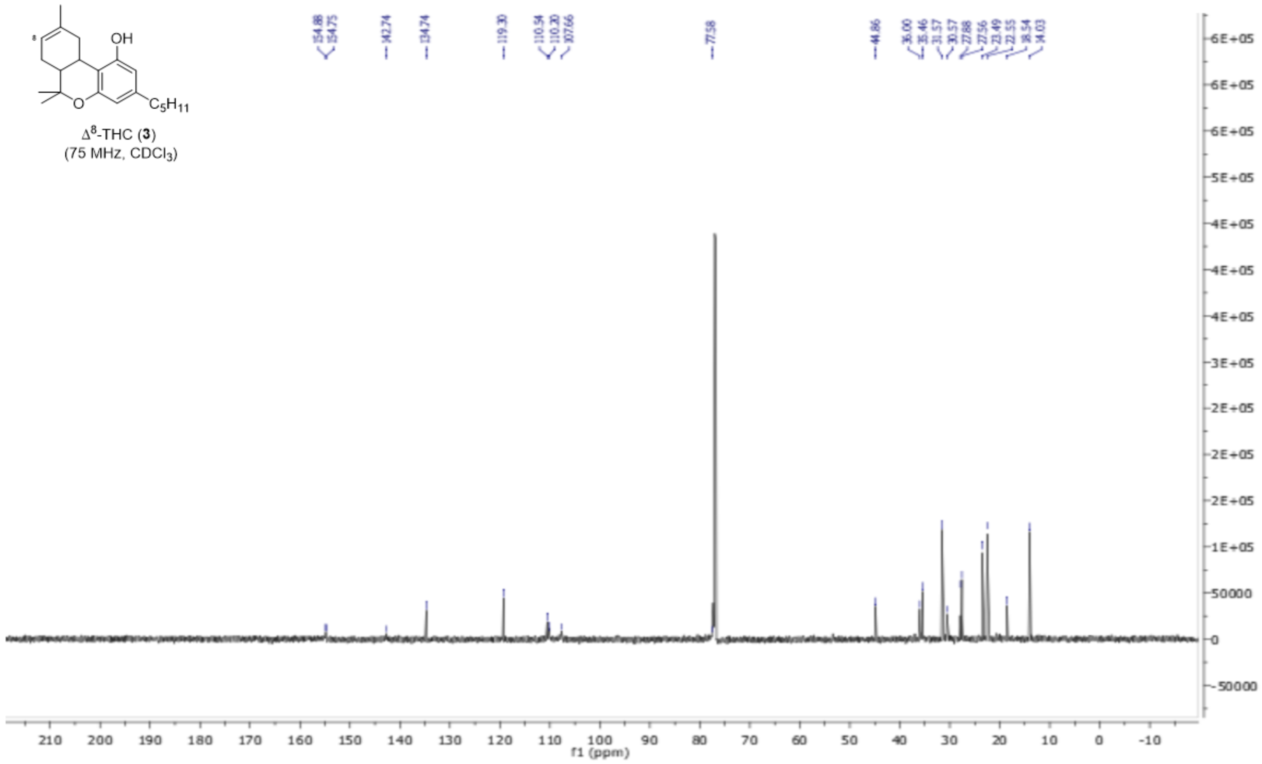

^{13}C{^{1}H} NMR Spectrum of *trans*-Δ^8-THC (**3**)

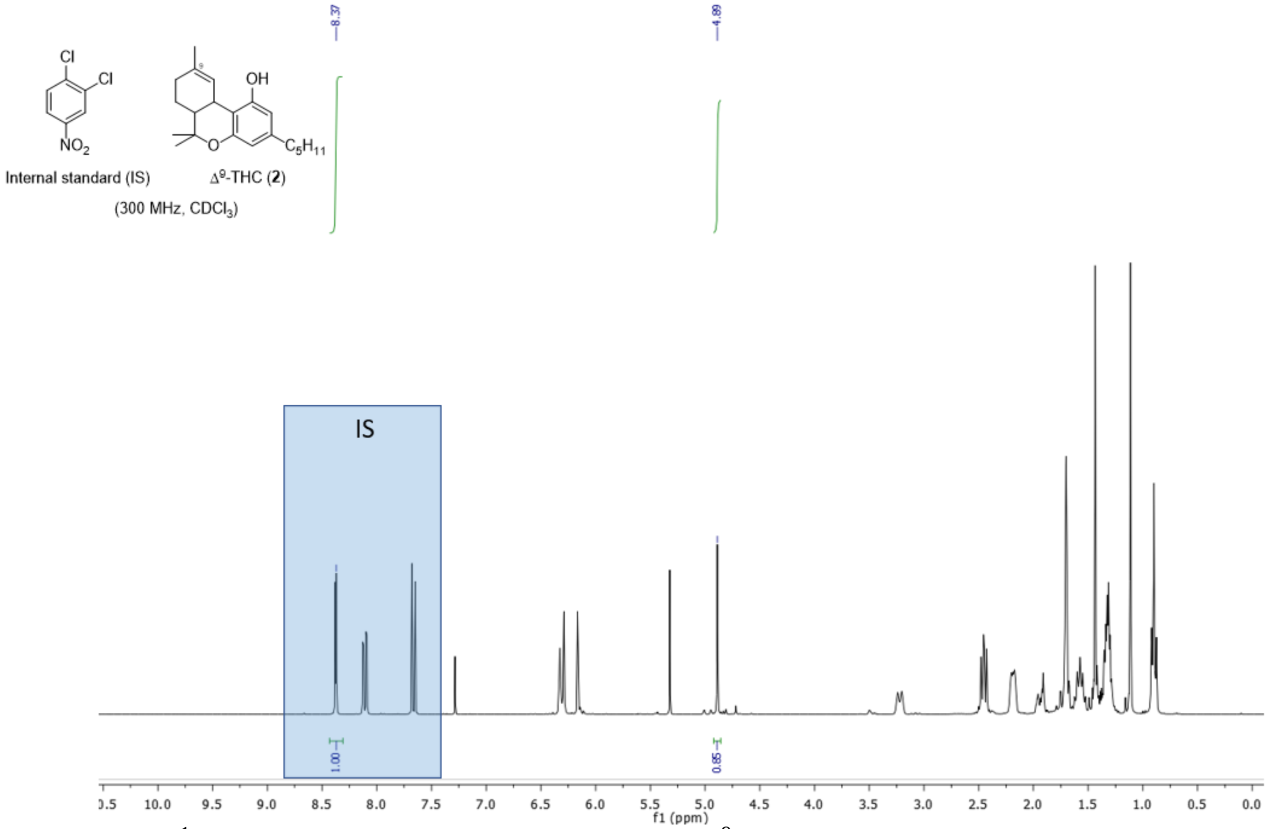

Example ^{1}H NMR Spectrum for reaction forming Δ^9-THC as product and using 1,2-dichloro-4-nitrobenzene as internal standard.

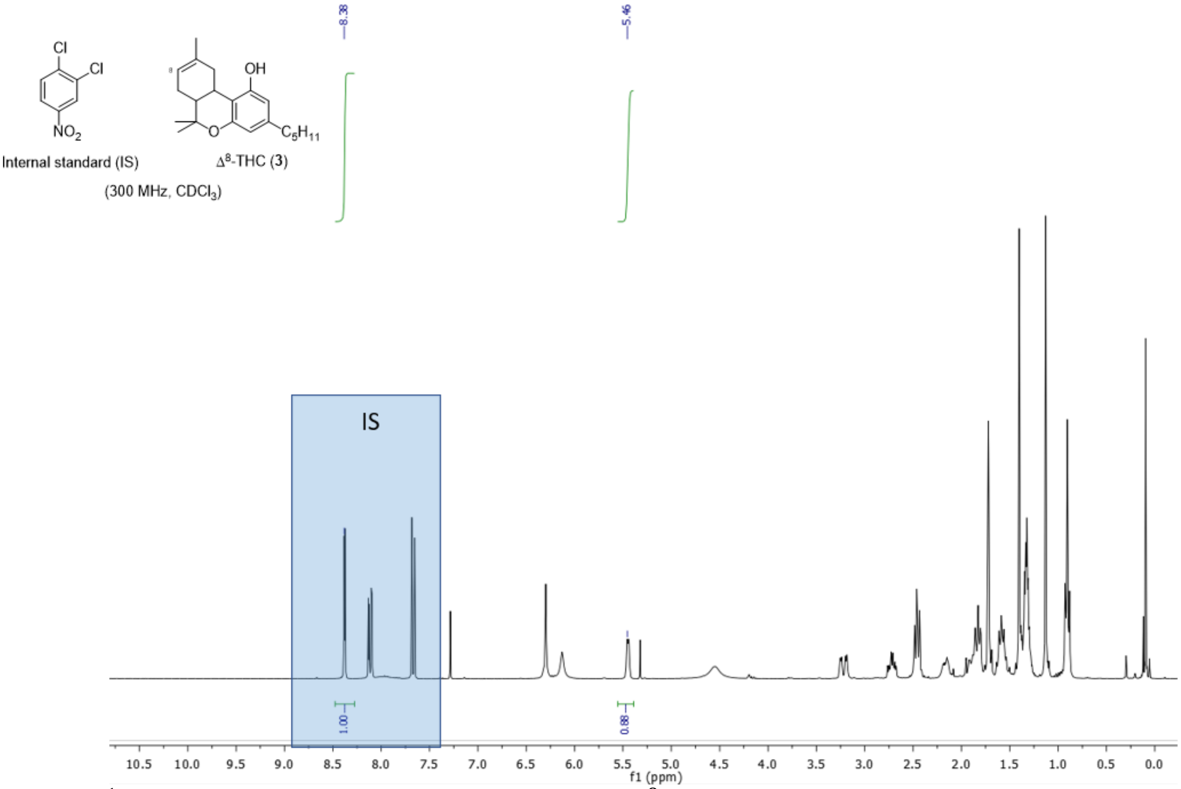

Example ^{1}H NMR Spectrum for reaction forming Δ^8-THC as product and using 1,2-dichloro-4-nitrobenzene as internal standard.
